# Pain in the penis due to attraction caused by neodymium magnets

**DOI:** 10.1002/ccr3.8141

**Published:** 2023-10-31

**Authors:** Yuki Akiyama, Ryo Ichibayashi

**Affiliations:** ^1^ Department of Orthopaedic Surgery Toho University Medical Center Sakura Hospital Chiba Japan; ^2^ Division of Emergency Medicine Department of Internal Medicine Toho University Medical Center Sakura Hospital Chiba Japan

**Keywords:** foreign body, magnet, neodymium, penis

## Abstract

Multiple neodymium magnets can pinch tissue and cause barotrauma. Be careful if the tissue of the penis or foreskin is pinched, as this may cause foreskin necrosis or damage to the urethra.

## 1 CLINICAL PICTURE

**FIGURE 1:**
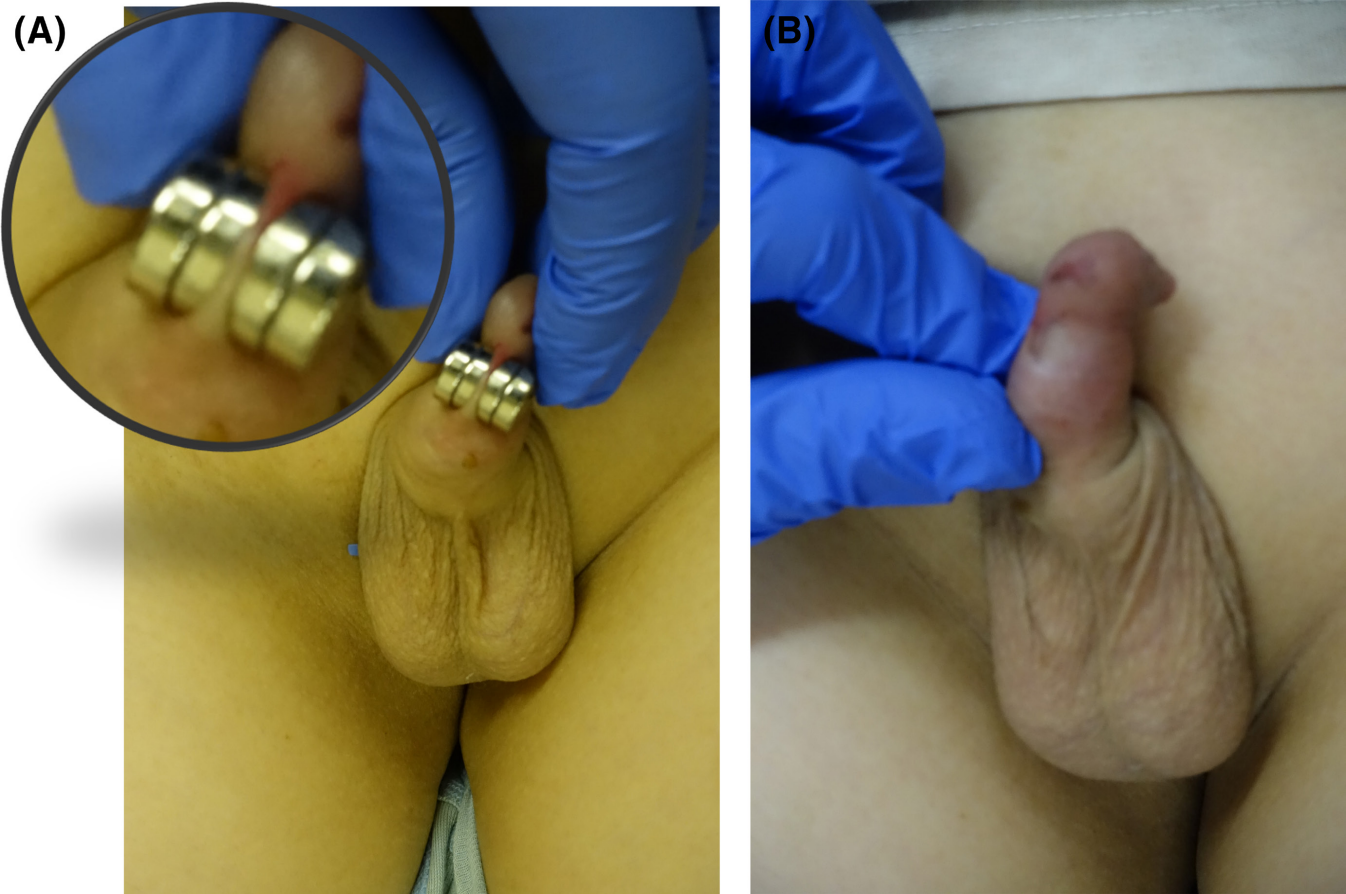
**(**A) Neodymium magnet that pinches the foreskin of the penis. (B) Foreskin with a crush injury.

An 11‐year‐old boy. He has no history of developmental disabilities or mental illness. The boy played with a neodymium magnet attached to his penis in the bathroom out of curiosity. However, the neodymium magnet remained attached to his penis and could not be removed, and he was transported to our hospital by ambulance with the chief complaint of penile pain. Emergency crews tried to remove it, but it was impossible because it was painful, and there were concerns that it might damage the foreskin. Two hours had passed since he was transferred to our hospital because he was refused admission by multiple medical institutions. At the time of his visit, his penis had two circular neodymium magnets, each approximately 1 cm in diameter, placed against the foreskin of his penis (Figure 1A). Although it was difficult to remove it by pulling it in the opposite direction, it was possible to remove it by shifting the ground surface. After the foreskin was released, there was a crushed wound, so ointment was applied (Figure 1B). After confirming that there was no problem with the color tone of his glans, he returned home. The next day, he returned to the outpatient clinic and confirmed that the color of his glans was standard and that he could urinate. Neodymium magnets were developed in Japan in 1982 and have become famous worldwide due to their effectiveness. It is said to be the strongest magnet currently in use. Neodymium magnets are used in various fields and children's toys.^1^ For this reason, accidents such as accidental ingestion by children and insertion into the urinary tract or anus due to sexual preference are problems in the medical field. Accidents caused by multiple neodymium magnets, especially when accidentally swallowed or inserted with a foreign object, are dangerous because they adhere to each other and pinch tissue, resulting in pressure necrosis.^2^ This case also had a crush injury to the foreskin. A month later, the scar remained on my foreskin. Barotrauma caused by neodymium magnets often involves damage to thin tissues, including the intestinal tract and mucous membranes. The structures of the corpus cavernosum and the corpus cavernosum of the urethra, which form the penis, are spongy and soft tissues. This tissue becomes rigid as it fills with blood. For this reason, if not only the foreskin but also the corpus cavernosum of the penis is pinched, there is a possibility that blood flow to the corpus cavernosum and damage to the urethra, erectile dysfunction, ejaculation abnormalities, changes in penis shape may occur. After removing the neodymium magnet, it is necessary to observe the color of the glans and foreskin and the state of urination. In the long term, it may be necessary to monitor for erectile dysfunction, ejaculation abnormalities, and changes in penis morphology.

## AUTHOR CONTRIBUTIONS

**Yuki Akiyama:** Writing – original draft; writing – review and editing. **Ryo Ichibayashi:** Supervision; writing – review and editing.

## FUNDING INFORMATION

The author(s) received no financial support for this article's research, authorship, and publication.

## CONFLICT OF INTEREST STATEMENT

The authors have no conflict of interest to disclose.

## CONSENT

Written informed consent was obtained from the patient to publish this report by the journal's patient consent policy.

## Data Availability

The data presented in this study are available on request from the corresponding author. The data are not publicly available due to privacy and ethical considerations.
